# Molecular network of HCC aggressiveness

**DOI:** 10.18632/oncoscience.194

**Published:** 2015-08-20

**Authors:** Sang Geon Kim, Mi Jeong Heo, Yoon Mee Yang

**Affiliations:** College of Pharmacy and Research Institute of Pharmaceutical Sciences, Seoul National University, Seoul, Korea

**Keywords:** Gα12, HCC, microRNA, EMT

Hepatocellular carcinoma (HCC) is one of the main causes of cancer-related mortality worldwide. It has a poor prognosis due to aggressive phenotype, and limited and insufficient treatment options. In HCCs, acquisition of epithelial mesenchymal transition (EMT) features may account for tumor aggressiveness and chemo-resistance. Many of G protein-coupled receptors (GPCRs) are overexpressed and mutated in tumor microenvironments, promoting tumor growth and metastasis. The heterotrimeric guanine nucleotide-binding proteins are responsible for pathophysiological processes by transducing signals from GPCRs to downstream effectors. With the accumulation of knowledge that overexpression of specific G proteins are present in tumors, there has been an increased interest in identifying the role of specific G protein subtypes in the regulation of tumor biology. Among them, Gα_12_ facilitates potent neoplastic transformation and change of cancer cell to a more aggressive phenotype. In recent studies, we found the overexpression of Gα_12_ in the patients with HCC and their poor survival rate [1, 2].

**Figure 1:**
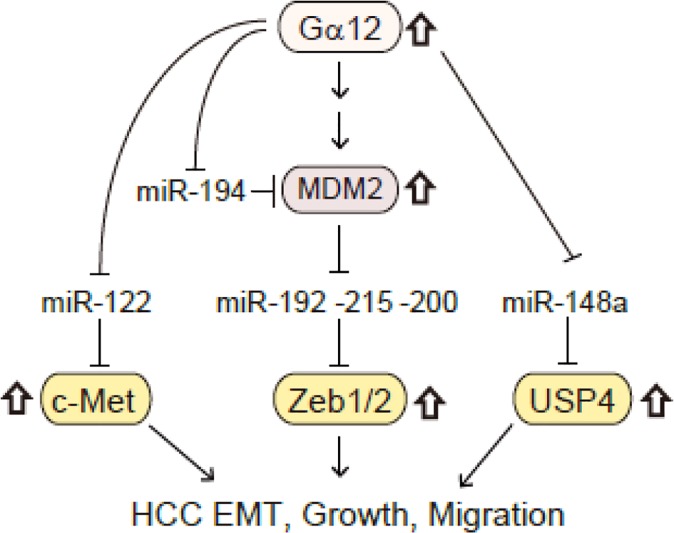
A schematic diagram illustrating the molecules responsible for HCC aggressiveness The patients with five positivities on Gα12, MDM2, c-Met, Zeb, and USP4 showed a significantly lower overall survival rate.

microRNAs (miRNAs) are small non-coding RNAs regulating gene expression and recently emerged as crucial players in hepatocarcinogenesis and HCC progression because of the ability to control multiple targets and modulate biological activities. In the previous study, we found that Gα_12_ overexpression causes dysregulation of a set of miRNAs necessary for the maintenance of epithelial phenotype [1]. One of the most interesting findings in our miRNome study was that Gα_12_ activation significantly repressed (<0.1-fold) all the liver-specific microRNAs (miR-122, miR-148, miR-192, and miR-194) defined by Barad et al. [3]. In our study, we showed that activated Gα_12_ down-regulated miRNAs (miR-200a/b, 194, and 192/215) increased by p53 except miR-200c. In HCC samples, MDM2 level increased in parallel with Gα_12_, and was inversely correlated with miR-200a/b levels (Figure 1). This event led to induction of Zeb1/2 and consequently EMT of HCC [1]. Other miRNAs regulated by Gα_12_ are also repressed in mesenchymal cancer cell type.

There is now growing evidence of crosstalk between GPCR and receptor tyrosine kinases (RTK): although GPCRs and RTKs are two different families of cell surface receptors, a majority of GPCRs transactivate RTKs and have growth-promoting activity. In a recent issue of Oncotarget, we reported that miR-122, a miRNA that accounts for 70% of total liver miRNAs, was most greatly decreased by Gα_12_ overexpression [2]. An integrative network of targets (putative or validated) of miR-122 showed that several RTKs, including c-Met, IGF1R, and FGFR2, were enriched interacting molecules of the pathway in cancer. Hence, Gα_12_ overexpression seems to enforce GPCR and/or RTK signaling cascades through miRNA dysregulation.

In another study, we found that dysregulation of miR-148a was associated with the poor prognosis of HCC and may account for the tumor progression to advanced stages [4]. Of the newly identified two targets of miR-148a (i.e., USP4 and S1P1), USP4 overexpression contributed to HCC progression toward more aggressive feature by facilitating TGF-β signaling pathways, growth advantage and migrating capability. In the same set of HCC samples, miR-148a dysregulation discriminated not only the overall survival and recurrence free survival rates of HCC, but the microvascular invasion. In this event, Gα_12_ overexpression appeared to contribute to miR-148a suppression [4].

Collectively, Gα_12_ signaling may affect MDM2, c-Met, Zeb, and USP4 expression, the key players of liver tumor EMT and growth advantage, mostly through dysregulation of the identified miRNAs (Figure 1). In an additional analysis, we found that the patients with five positivities on Gα_12_, MDM2, c-Met, Zeb, and USP4 in HCC showed a significantly lower overall survival rate as compared to others, providing an insight into the contribution of the molecular network to HCC malignancy.

The patients with four or less positivities exhibited moderate survival rates, whereas those with no positivity showed 100% survival over the period examined (>5 years). Our findings provide a key information on (1) the crosstalk of GPCR-Gα_12_-RTK receptors or enforcement of GPCR-G protein signaling through a positive feedback loop, and (2) the impact of the identified molecular network on the progression of HCC to more aggressive phenotype, supporting the concept that intervention of the molecules in the network may be of help to slow invasion and improve targeted therapy. From a clinical perspective, the molecules identified in the present study may be utilized as prognostic biomarkers to predict recurrence of HCC.
